# Resource efficient recovery of critical and precious metals from waste silicon PV panel recycling

**DOI:** 10.1016/j.wasman.2019.04.059

**Published:** 2019-05-15

**Authors:** Fulvio Ardente, Cynthia E.L. Latunussa, Gian Andrea Blengini

**Affiliations:** a European Commission, Joint Research Centre (JRC), Ispra, Italy; b Politecnico di Torino, Corso Duca degli Abruzzi 24, 10129 Torino, TO, Italy

**Keywords:** Critical raw material (CRM), Secondary raw material (SRM), Crystalline silicon photovoltaic panel (c-Si PV), Life Cycle Assessment (LCA), E-waste, Waste Electrical and Electronic Equipment (WEEE), ADP, Abiotic Depletion Potential, CED, Cumulative Energy Demand, CRM, Critical Raw Materials, c-Si PV, crystalline silicon photovoltaic, Cd-Te PV, cadmium-tellurium photovoltaic, EC, European Commission, EoL, end-of-life, EU, European Union, FRELP, Full Recovery End-of-Life Photovoltaic process, HF, hydrogen fluoride, KPK, Kynar-polyethylene terephthalate-Kynar, IEA, International Energy Agency, ILCD, International reference Life Cycle Data system, LCA, Life Cycle Assessment, LCIA, life cycle impact assessment, PPE, polyethylene terephthalate-polyethylene terephthalate-ethylene vinyl acetate, PV, photovoltaic, SRM, secondary raw materials, TPT, Tedlar-polyethylene terephthalate-Tedlar, VOC, Volatile Organic Compound, WEEE, Waste Electrical and Electronic Equipment

## Abstract

Although the amount of waste photovoltaic (PV) panels is expected to grow exponentially in the next decades, little research on the resource efficiency of their recycling has been conducted so far. The article analyses the performance of different processes for the recycling of crystalline silicon PV waste, in a life cycle perspective. The life cycle impacts of the recycling are compared, under different scenarios, to the environmental benefits of secondary raw materials recovered. Base-case recycling has a low efficiency and, in some cases, not even in line with legislative targets. Conversely, high-efficient recycling can meet these targets and allows to recover high quality materials (as silicon, glass and silver) that are generally lost in base-case recycling. The benefits due to the recovery of these materials counterbalance the larger impacts of the high-efficiency recycling process. Considering the full life cycle of the panel, the energy produced by the panel grants the most significant environmental benefits. However, benefits due to high-efficient recycling are relevant for some impact categories, especially for the resource depletion indicator. The article also points out that thermal treatments are generally necessary to grant the high efficiency in the recycling. Nevertheless, these treatments have to be carefully assessed since they can be responsible for the emissions of air pollutants (as hydrogen fluoride potentially released from the combustion of halogenated plastics in the panel’s backsheet). The article also identifies and assesses potential modifications to the high-efficiency recycling process, including the delocalisation of some treatments for the optimisation of waste transport and the introduction of pyrolysis in the thermal processing of the waste. Finally, recommendations for product designers, recyclers and policymakers are discussed, in order to improve the resource efficiency of future PV panels.

## Highlights

• Resource efficiency of PV recycling processes is analysed in different scenarios.
• Processes with high-efficiency can recycle up to 83% of the waste panel.
• Obtaining high-quality recycled materials from PV grants significant benefits.
• The treatment of halogenated plastics represents a bottleneck for PV recycling.
• Knowing the composition of panels is essential to boost their efficient recycling.

## 1 Introduction

The exponential growth in photovoltaic (PV) panel waste is expected to result in an increase from 100 000 tonnes in 2016 to 60–70 million tonnes in 2050 (Weckend et al., 2016, Statista, 2018).

Almost two decades ago, Fthenakis (2000) highlighted the importance of investigating the technical and environmental benefits of PV waste recycling. Recycling systems for PV waste and regulatory schemes for their end-of-life (EoL) management have only recently emerged (Weckend et al., 2016). According to Bilimoria and Defrenne (2013), the collection and recycling of PV waste have been much lower than expected, representing only 10% of potential PV waste volumes. The low volumes of PV waste recovered and the uneven development of the recycling technologies could be explained by several factors. Firstly, the lifetime of panels has turned out to be generally longer than estimated. Some experiments proved that the rate of degradation in the efficiency of crystalline silicon photovoltaic (c-Si PV) panels was around 0.5% per year, much lower than the initially estimated loss of 1% per year (Jordan and Kurtz, 2012). Not surprisingly, customers are inclined to keep PV panels operative even with a lower efficiency, instead of disposing of them or replacing with new ones. Consequently, a longer lifetime of panel postpones the generation of PV waste and the follow-up.

Secondly, the profitability of current recycling is low as it is based on the recovery of few materials, such as aluminium, glass and copper (Choi and Fthenakis, 2014, D’Adamo et al., 2017, Cucchiella et al., 2015). This low profitability has negatively affected investment in the collection and treatment of PV waste. Profitability could be boosted by an increase in the number, volume and purity of recycled materials (including e.g. silver and silicon) (Peeters et al., 2017, International Energy Agency (IEA), 2018).

Moreover, the management of PV waste is complex and influenced by legal obligations and the attitudes of users. The European Community Directive on Waste Electrical and Electronic Equipment (WEEE) represents the first example worldwide of regulation on PV waste (EC, 2012). This Directive pushes for higher resource efficiency in recycling and better design-for-recycling for new panels.

Consumer behaviour around the purchase and use of PV panels has been investigated by various authors (Haas et al., 1999, Faiers and Neame, 2006, Jager, 2006, Keirstead, 2007). However, consumer attitude towards PV disposal remains largely unexplored. Nevertheless, improper management of PV waste can cause the loss of valuable resources and the dispersion of potentially hazardous substances contained in the panels. The low amount of PV waste collected discourages the development of new technologies for PV recycling (IEA, 2017), since a sufficiently large and stable flow of input waste is essential to sustain a recycling business (Hagelüken, 2012). On the other hand, this is an opportunity for researchers, since the sector has not been sufficiently explored and the efficiency of the recycling processes still shows large margins for improvement.

PV technologies largely rely on the availability of various materials, including silicon. The demand for silicon for the PV sector in the European Union (EU) is expected to rise from 33 kilotonnes (kt) in 2015 to 235 kt in 2030 (EC, 2018). The high economic importance of silicon for Europe, together with the high supply risk, justified its inclusion in the list of critical raw materials (CRMs) for the EU (EC, 2017). The European Commission (EC) recognised the need for actions to foster resource-efficient solutions to recover silicon and other materials from PV, to reduce its criticality and overall to improve the circularity of the European economy (EC, 2018). Resource efficiency here means using the world’s limited resources in a sustainable manner, while minimising impacts on the environment (Weckend et al., 2016).

Improper collection and/or disposal of PV waste entails the loss of valuable resources and the dispersion of potentially hazardous substances contained in the panels. This article aims to assess the resource efficiency of recycling processes for c-Si PV waste by following a life cycle approach with multiple objectives. In particular, efficient recycling of waste allows the production of secondary raw materials (SRMs), meaning materials recycled from waste that can be injected back into the economy (RMIS, 2018). In a life cycle perspective, the article analyses environmental benefits from recycling and weighs them against the burdens of the processing, with an emphasis on the value of recycled materials compared to the impacts of the manufacturing and use of the panels.

## 2 Literature review on EoL treatments of PV panels

Life Cycle Assessment (LCA) methodology and underlying Life Cycle Impact Assessment (LCIA) methods have been considered as a good basis for screening the resource efficiency of product systems (Schneider et al., 2016, Huysman et al., 2015), including specific application to recycling processes (Birat, 2016, Sfez et al., 2017). LCA has been widely adopted to assess the environmental performance of PV panels, as illustrated by several reviews (Sherwani et al., 2010, Gerbinet et al., 2014, Peng et al., 2013). Although EoL represents a crucial phase in the life cycle of products, the fate of PV waste in LCA studies has been largely excluded or neglected by researchers (Gerbinet et al., 2014, Sherwani et al., 2010). Latunussa et al. (2016) identified various reasons behind the exclusion of EoL from the LCA of PV technologies, including the limited number of detailed studies on recycling processes for waste panels, and the consequent lack of primary data as input for the life cycle inventory phase. In addition, despite high potential interest by researchers in PV recycling, primary data about PV recycling are still very scarce.

Some EoL aspects of PV panels have been discussed by Müller et al. (2005), which analysed the Deutsche Solar process for cell reuse; Zhong et al. (2011), which provided some details on material recovery from PV recycling; and Held and Ilg (2011), which analysed the First Solar recycling process for CdTe PV modules. A recent report by the International Energy Agency (IEA) highlighted the potential relevance of the EoL management of PV panels, since their recycling could unlock a large stock of valuable raw materials, including CRMs (Weckend et al., 2016). Additional interest in PV recycling has also been demonstrated by the recent high number of patents developed to eliminate the encapsulant from the laminated structure of the PV, this being one of the most difficult and important targets for PV recycling technologies (IEA, 2018).

Only in recent years have some studies focused on dedicated application of the LCA to the analysis of recycling processes for c-Si PV. For example, Held (2013) analysed the potential benefits and impacts of the recovery of glass and bulk metals (ferrous metals, aluminium and copper), and concluded that the recycling of these materials can reduce the life cycle impacts of PV modules by around 4%–11%.

In a previous study (Latunussa et al., 2016), we investigated a novel process for the recycling of c-Si PV waste panels - Full Recovery End-of-Life Photovoltaic (FRELP). In that article we provided a detailed analysis of PV recycling with an estimation of the related life cycle impacts. However, Latunussa et al. (2016) did not estimate potential benefits using a full life cycle perspective, or explore measures to improve the efficiency of the recycling process.

An initial review of life cycle inventory data for PV recycling has been provided by Wambach (2017), based on 16 recyclers worldwide contacted in 2015 and 2016. Wambach (2017) also provided the ranges of recycling yields for different materials in different processes (e.g. glass 59%−75%; non-ferrous metals 13.5%−21.8%). The study concluded that ‘no detailed statistics are currently available regarding the type and vintage of modules processed in recycling facilities’ with still ‘little interest in detailed assessments of recycling process inputs, likely because such additional efforts are currently neither mandatory nor remunerated’.

A first example of comprehensive assessment of material recovery and life cycle impacts for PV recycling has only recently been presented by Corcelli et al. (2018). They applied the LCA to the analysis of two different recycling routes for c-Si PV panels and assessed the environmental benefits of SRM production. They based the analysis on laboratory tests conducted under optimal conditions for various PV samples. Corcelli et al. (2018) concluded that a well-designed recovery process must focus on all high-value materials, such as silicon and silver. Nevertheless, scale-up from laboratory to full-scale industrial process would be necessary to confirm the findings.

Interestingly, studies by Latunussa et al., 2016, Wambach, 2017, Aryan et al. (2018) and Corcelli et al. (2018) agree on the environmental significance of incinerating the halogenated plastics in the backsheet. Unfortunately, as reported by Wambach (2017), if the ‘halogen content is too high, then incineration in a specialised hazardous waste plant must be carried out’, in order to minimise the emission of potentially toxic air pollutants such as hydrogen fluoride (HF). Moreover, the above-mentioned studies provided little information on the impacts of incinerating PV plastics. For example, Corcelli et al. (2018) estimated that the incineration of waste panels releases 0.87 g/m^2^ of HF, while Latunussa et al. (2016) considered life cycle inventory data for general plastic incineration.

Detailed research on the thermal treatment of plastics in PV backsheet is presented by Fraunhofer, 2017, Aryan et al., 2018, supported by experimental results and primary industry data. Three types of backsheet have been analysed, including two fluorinated plastics (Tedlar-polyethylene terephthalate-Tedlar – TPT, and Kynar-polyethylene terephthalate-Kynar – KPK), and a fluorine-free backsheet (polyethylene terephthalate-polyethylene terephthalate-ethylene vinyl acetate – PPE). Fraunhofer (2017) measured that the fluorine content of backsheet can be up to 9% (in weight). Aryan et al. (2018) recently published an LCA of different EoL treatment pathways for PV backsheets. However, the studies by Fraunhofer (2017) and Aryan et al. (2018) focused on the treatment of the backsheets and did not investigate the impact over the full life cycle of the PV panel.

It is thus necessary to optimise the efficiency of waste recycling (and to minimise the related impacts) to claim the sustainability of PV technologies. However, LCA practitioners have only recently showed a growing interest in assessment of the EoL of PV technologies. Assessment of the resource efficiency of PV recycling remains largely unexplored, especially concerning the benefits of increasing recovery rates for different materials in PV waste.

## 3 Materials and method

From a life cycle perspective, resource efficiency is assessed by accounting for the impacts and benefits of c-Si PV waste recycling according to LCA methodology (ISO, 2006). The functional unit is the ‘recycling of 1000 kg of c-Si PV waste panel with TPT (1) backsheet’. The life cycle inventory data for the recycling refer to the FRELP process, as presented in a previous publication (Latunussa et al., 2016). Compared to the previous research, the present article is characterised by several new features.

(a) The life cycle inventory data (2) for the FRELP recycling process have been complemented with primary experimental data from Fraunhofer (2017) on the emission of air pollutants during incineration of the panel’s backsheet (as detailed in Section 4.1.4). Moreover, two additional impact categories have been added to the LCIA (3) in order to capture relevant air emissions (e.g. HF) derived from incineration of the backsheet: ‘acidification’ impact from the EDIP2003 method (Hauschild and Potting, 2005) and ‘human toxicity’ from the ReCiPe method (Goedkoop et al., 2013).
(b) The system boundaries have been extended to the production of SRMs, with a focus on the recovery of precious metals and CRMs (Section 4), in order to assess whether and how far potential environmental benefits from recycling may exceed the burdens of the waste processing.
(c) The FRELP recycling process has been compared to base-case recycling practices (‘baseline processes’) as used in European WEEE recycling plants (Section 4.1.3). The article analyses, from a life cycle perspective, the importance of recycled materials compared to the impacts of the manufacturing and operation of the panels.
(d) The resource efficiency of FRELP has also been assessed in comparison with impacts and benefits related to the manufacturing and operation stages of the PV panel (Section 4.2).
(e) Potential modifications to the FRELP process have been identified and assessed, including the delocalisation of some treatments for the optimisation of waste transport (Section 4.3.1), and the introduction of alternative pyrolysis processing for the PV backsheet (Section 4.3.2).
(f) Finally, recommendations for product designers, recyclers and policymakers are discussed, in order to improve the resource efficiency of future PV panels.

## 4 Results and discussion

The resource efficiency of the PV recycling processes is assessed through various scenarios. Initially, two scenarios are considered; these are then expanded from a life cycle perspective and compared to benefits and impacts related to PV manufacturing and operation stages. Finally, Section 4.3 analyses two additional scenarios, in which modifications to the recycling process are introduced to reduce the overall environmental impacts.

### 4.1 Environmental impacts and benefits of PV recycling processes

Two PV recycling scenarios are assessed: innovative high-efficiency recycling (represented by the FRELP process, as we presented in Latunussa et al. (2016)) and a ‘baseline’ process, representative of average PV recycling practices in the EU. The two scenarios are discussed and compared in Section 4.1. Section 4.1.1 presents an additional analysis of the treatment of panels with different backsheets.

#### 4.1.1 High-efficiency PV recycling process

**Fig. 1:**
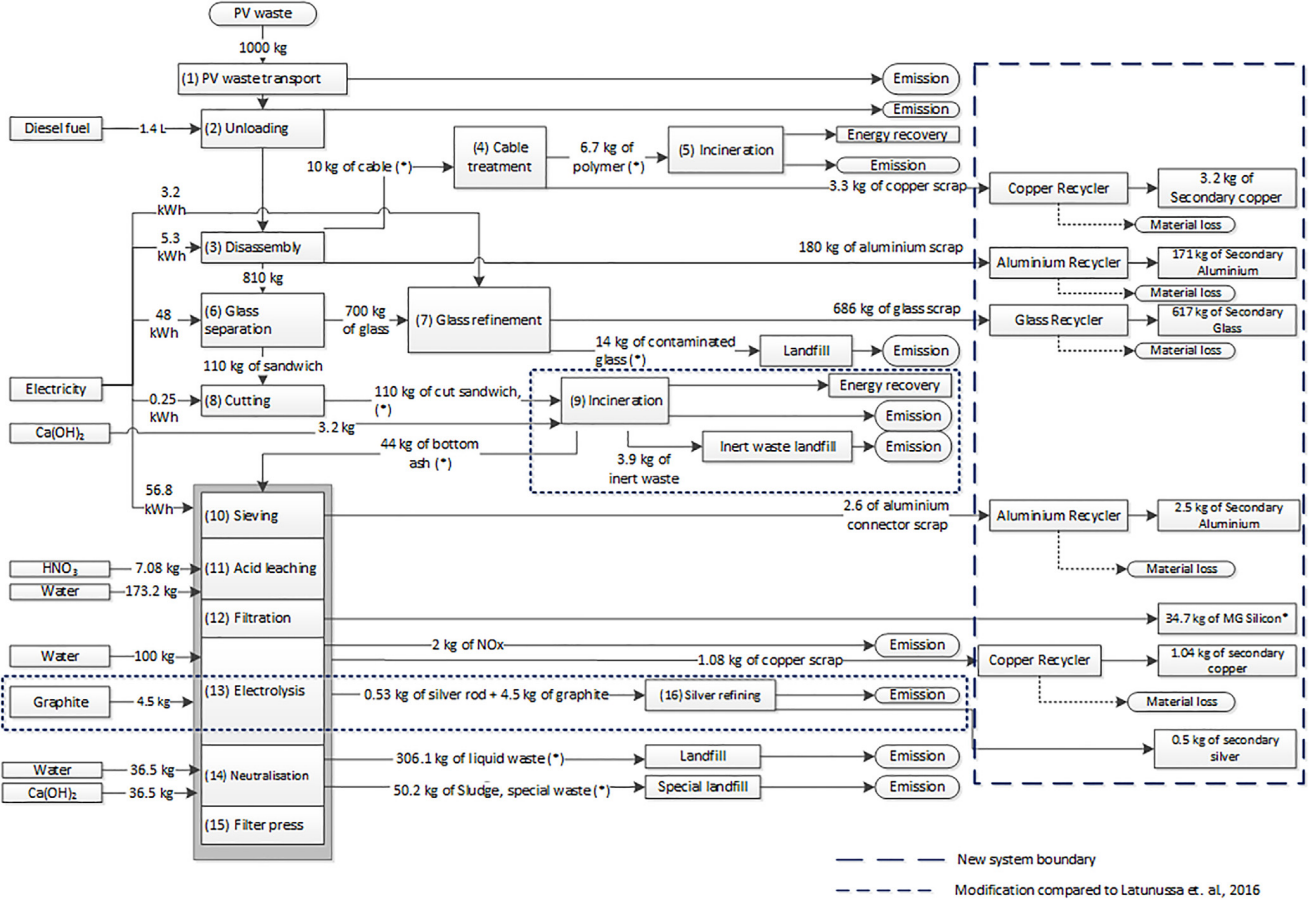
Input and output flows for FRELP recycling process for c-Si PV waste (with dashed parts as modified from Latunussa et al., 2016). Transport between the processes is highlighted with an asterisk (*).

The FRELP recycling process for c-Si PV panels has been acknowledged as one of the most advanced processes currently developed worldwide in terms of material recovery from PV waste (Wambach, 2017). This was developed by SASIL (2015) up to a pilot phase, and considered ready for full application at industrial stage. The key steps in the process are illustrated in Fig. 1. After transport (1), the PV waste is unloaded (2) and transferred into an automated system for PV dismantling (3), to remove the frames and cables, which are further treated for copper recycling and energy recovery of plastics (4 and 5). The waste panels then undergo a glass separation process (6), in which the glass layer is detached from the remaining layers of polymers and cells (the ‘PV sandwich’). The glass scraps are channelled to a refinement process (7), while the PV sandwich is reduced in size (8) and later treated in an incineration plant (9). Ashes from the incineration are sieved (10) and treated by acid leaching (11). The acid solution is then filtered (12) (to recover the silicon), and treated with electrolysis (13) (to recover silver and copper). The residues from the electrolysis are subsequently neutralised (14) and filtered (15). Silver is separated by electrolysis on graphite rods, which is finally burnt to liberate silver (16).

Fig. 1 also shows the energy and material flows for each step of the process, including the differences from the previous analysis in Latunussa et al. (2016) as evidenced by dashed boxes. In particular, data on heating values of the backsheets and emissions during the incineration process (step 9) have been derived from Fraunhofer (2017). Inputs of graphite rods have been added to the process (SASIL, 2015). System boundaries for the analysis have been also enlarged to include the amount of SRMs produced by the process (4). The estimated recycling rate for the process is 83%. Plastics in cables, encapsulations and backsheet are intended to be further incinerated with energy recovery.

#### 4.1.2 Baseline PV recycling process

**Fig. 2:**
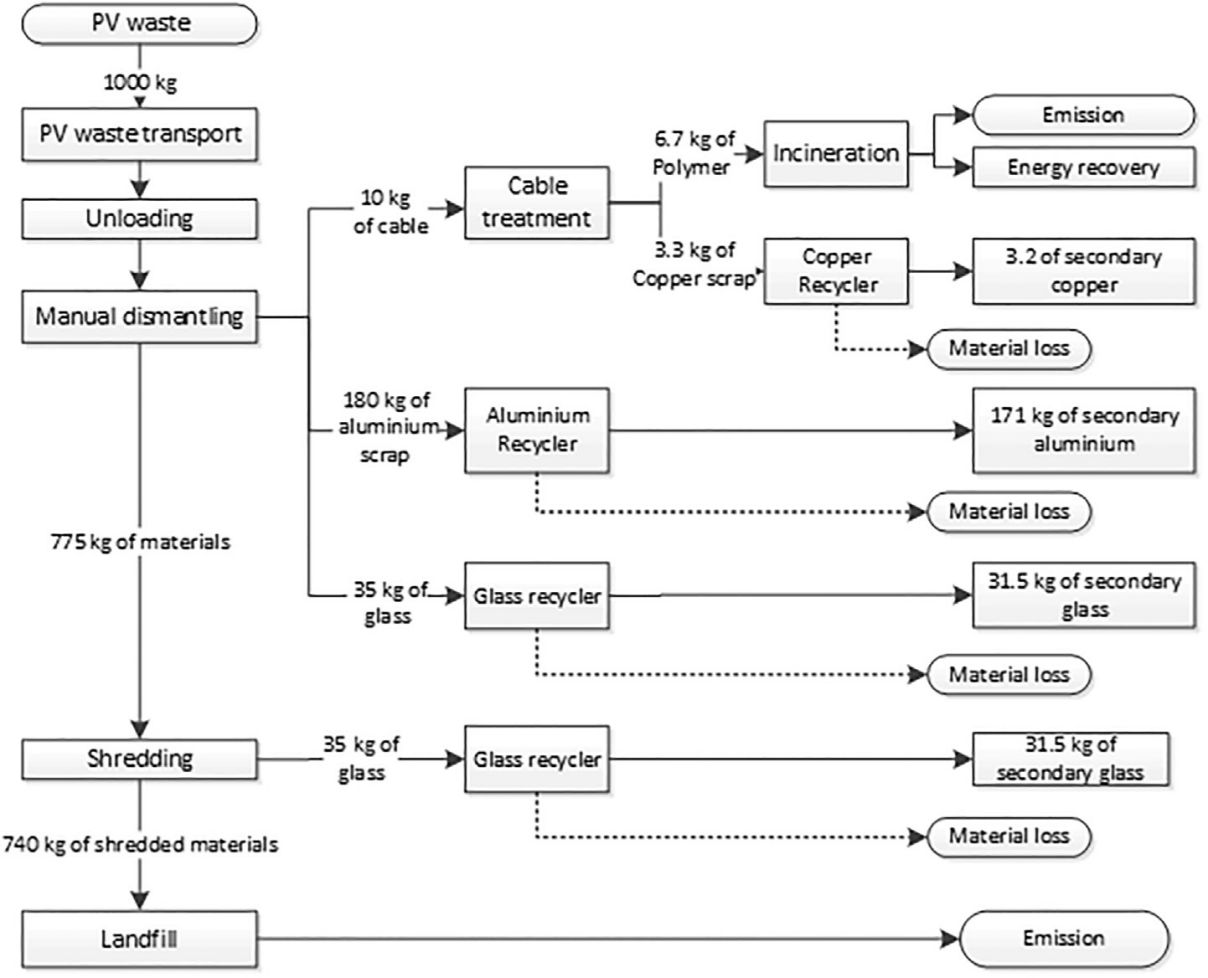
Input and output flows for a baseline recycling process for c-Si PV waste.

The baseline recycling scenario is considered representative of average practices in Western European WEEE recycling plants not equipped with specialised technologies for PV recycling. This scenario has been built based on visits we made to two recycling plants (in Italy and Spain), complemented by information available in the literature (Zhong et al., 2011, Stolz and Frischknecht, 2016). We observed that recycling operators start with manual dismantling of the panel’s frames and cables, which are subsequently sorted for recycling. The remaining parts of the panel are then treated with simple techniques (e.g. hammered or ground to partially separate the glass) or directly shredded with other WEEE. Due to the heterogeneity of the PV panel (including glass, encapsulations, silicon cells and multi-polymer backsheet), this process is not able to efficiently separate different materials. We estimate that the baseline process can separate up to 10% of the glass, whereas PV cells and plastics are landfilled with residuals from shredding (5). Fig. 2 illustrates the material flows for the baseline PV recycling process. The overall recycling rate achieved by such processes is around 24%, well below the current minimum target of 80% (in mass) of reuse and recycling, as set by the WEEE Directive.

#### 4.1.3 Comparison of the two recycling processes studied

**Fig. 3:**
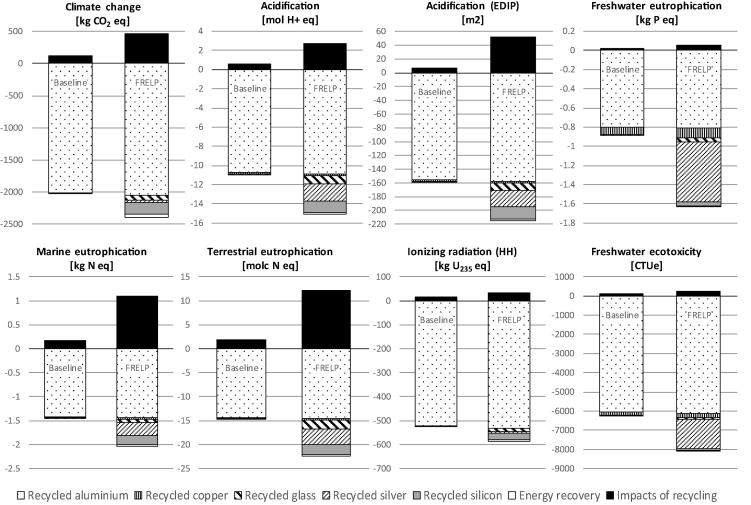
Impact assessment (Part 1): Comparison of FRELP process with baseline recycling process for c-Si PV waste (with TPT backsheet).

**Fig. 4:**
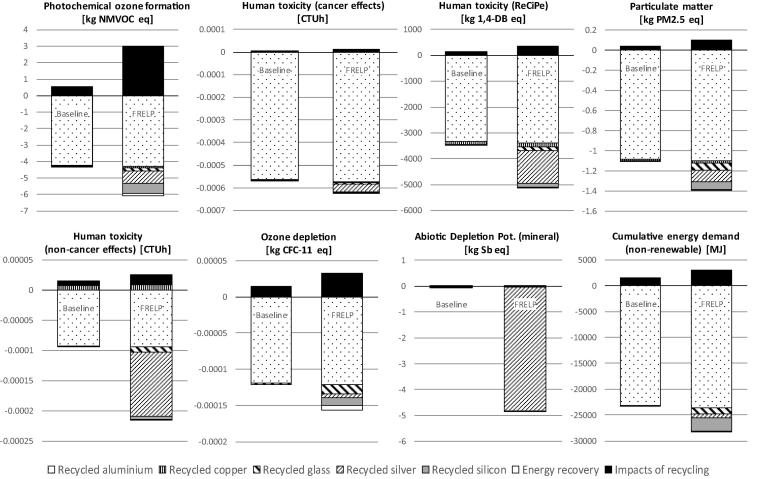
Impact assessment (Part 2): Comparison of FRELP process with baseline recycling process for c-Si PV waste (with TPT backsheet).

In the comparison with the FRELP process, the impacts of the baseline recycling are estimated assuming: waste is transported a distance of 100 km; dismantling is done manually and does not imply any energy consumption or other impacts; electricity consumption for shredding refers to an average shredder for WEEE; unsorted shredded fractions are sent to landfill (including 100 km transport). Life cycle inventory data for electricity (European mix), transport (low-capacity lorry) and landfill (for inert materials) refer to the Ecoinvent (2007) database. Fig. 3, Fig. 4 illustrate the LCIA results. The environmental benefits related to SRMs are accounted as benefits due to the avoidance of using primary raw materials, at the net of the impacts for the production of the SRMs (Ardente and Mathieux, 2014). This assessment takes into account the effective quantity of SRMs produced (net of losses), the raw material potentially substituted by the SRMs, and inventory data for primary and secondary materials. The analysis of SRMs derived from the processes, and of primary materials potentially substituted, has been performed jointly with the developer of the FRELP process. Table 1 presents a summary of the main features of materials recycling in the FRELP and baseline processes.

**Table 1:** Summary of main features related to materials recycling in the FRELP and baseline processes.

Material	Recycling rate [%]		Detail of material output		Potentially substituted material	
	Baseline	FRELP	Baseline	FRELP	Baseline	FRELP
Aluminium	92%	94%	Aluminium scraps from panel frames, separated by manual dismantling	Aluminium scraps from frames and internal connectors, separated to high purity by automated dismantling and further processed	Secondary aluminium, equivalent in quality to primary material	Secondary aluminium, equivalent in quality to primary material
Copper	72%	90%	Copper scraps from cables	Copper scraps from cables and from interior parts of the PV panel	Secondary copper, equivalent to primary material	Secondary copper, equivalent to primary material
Glass	9%	88%	Glass scraps	Glass scraps separated through a highly selective process in order to maintain high purity. Antimony in glass is assumed to be lost.	Glass for medium-low quality applications	Glass for medium-high quality applications (e.g. production of flat glass).
Silicon	–	95%	–	Silicon separated by acid leaching to obtain high purity SRM	–	Metallurgical grade silicon metal
Silver	–	94%	–	Silver separated by electrolysis on graphite rods	–	Secondary silver equivalent to primary material

The results showed that the impacts of the FRELP recycling process (Fig. 3, Fig. 4) are higher than those of the baseline process. FRELP is in fact a more complex and energy-intensive process. Nevertheless, the greater recovery of high-purity secondary materials and energy through FRELP corresponds to higher benefits for all the impact categories considered. FRELP is observed to have better performance in terms of net benefits (i.e. benefits minus impacts) for the majority of the impact categories. For example, in relation to climate change, the FRELP process represents an impact of 461 kg CO_2_ equivalent (CO_2_ eq) per tonne of treated waste (compared to 116 kg CO_2_ eq/tonne for the baseline process). On the other hand, FRELP allows a saving of 2 400 kg CO_2_ eq/tonne, compared to 2 025 kg CO_2_ eq/tonne for the baseline.

FRELP resulted in lower net benefits for eutrophication (marine and terrestrial) and photochemical ozone formation impacts.

The benefits of the baseline process are also high for several impact categories, as the recycling of the aluminium frames alone brings large environmental gains. The benefits of the two processes are indeed very similar for certain impact categories (e.g. climate change and cumulative energy demand – CED), but they differ substantially for some other categories (e.g. abiotic depletion potential – ADP). This major difference is mainly related to silver, which is recycled in FRELP but lost in the baseline.

Concerning generation of SRMs, aluminium is the most significant in terms of environmental benefits for several impact categories. Recycled silver and silicon are also important for various impact categories. In particular, recycled silver has significant benefits for ADP and freshwater eutrophication. The benefits from recycled silicon range from 1% to 10% for the different impact categories (e.g. 7% of overall benefits for climate change).

#### 4.1.4 Analysis of the incineration of PV panels with different backsheets

As mentioned in Section 3, the impact categories recommended by Latunussa et al. (2016) were not sensitive to HF emissions. This justified the inclusion of two additional impact categories related to acidification (EDIP) and human toxicity (ReCiPe). Fig. 3, Fig. 4 show that incineration of the PV sandwich (with TPT backsheet) causes the emission of HF, which is responsible for about 40% of the acidification impact (EDIP method, Fig. 3) and about 80% of the human toxicity impact (ReCiPe method, Fig. 4). Due to the magnitude of these emissions, we decided to further investigate the impact assessment for the incineration step.

The potential fluorine content in PV panels represented a bottleneck for the FRELP process. Indeed, incineration of fluorinated plastics can cause emissions to air of HF, a pollutant potentially responsible for acute toxicity effects (Stavert et al., 1991). Therefore, FRELP originally anticipated the use of an advanced incineration plant (step 9 in Fig. 1). However, in the previous analysis discussed in Latunussa et al. (2016), we did not focus on the specific impacts while considering average life cycle inventory data for general plastic incineration.

In this section, we analyse a new scenario for the treatment of PV panels with three different backsheets, including two fluorinated plastics (TPT and KPK) and a fluorine-free plastic (PPE) (6). The treatment of these PV wastes is assumed based on the FRELP process, as described in Section 4.1.1, with the exception of the impact during incineration (i.e. step 9 in Fig. 1).

Information on the release of HF from fluorinated plastics in the backsheet has been derived from interviews we conducted with a manufacturer of PV backsheet, who also sponsored the collection and analysis of experimental data (Fraunhofer, 2017). Fraunhofer (2017) measured that the fluorine in the backsheet is fully released to air when plastics are incinerated at 750 °C or above.

We assumed that these HF emissions could be reduced by 80% in incineration plans equipped with dedicated abatement systems (Biganzoli et al., 2015). In assessing incineration, we also considered the use of energy and reagents for the abatement (data derived from Ecoinvent (2007)). CO_2_ emissions from the incineration of both fluorinated and fluorine-free waste have been considered assuming complete combustion of all the carbon content in the polymers. Incineration of the ethylene-vinyl-acetate in the backsheet can be responsible for the emission of volatile organic compounds (VOCs) (Hull et al., 2002). However, these emissions were not considered due to the lack of information. Other air emissions from incineration have been extrapolated from average life cycle inventory data in the literature on the incineration of generic plastic waste (Latunussa et al., 2016).

Table 2 shows the impact assessment for the recycling of PV panels with different backsheets. Compared to the treatment of waste with TPT backsheet, the FRELP process applied to fluorine-free PV waste is characterised by lower impacts for acidification (EDIP; 40% lower), human toxicity (ReCiPe; 14% lower), ozone depletion and CED (1% lower). These results are due to the avoidance of HF emissions and, to a lower extent, the avoided use of reagents for the abatement of acid emissions. The differences for the other impact categories are however negligible. It is interesting to note that incineration of the fluorine-free PV waste has a slightly higher climate change impact, due to the higher carbon content in PPE. The incineration of waste with KPK always has the highest impacts, due to the higher fluorine content in the backsheet.

**Table 2:** Comparison of the impacts of FRELP process, for PV panels with different backsheets, and detail of incineration (step 9).

Impact category	Unit	Incineration (FRELP step 9)			FRELP (full process)		
		KPK	PPE	TPT	KPK	PPE	TPT
Climate change	[kg CO_2_ eq]	1492.5	1489.7	1477.6	462.7	462.4	461.0
Ozone depletion	[kg CFC-11 eq]	4.4E-06	1.1E-06	3.1E-06	3.3E-05	3.2E-05	3.3E-05
Human toxicity, cancer effects	[CTUh]	1.4E-06	1.2E-06	1.3E-06	1.4E-05	1.4E-05	1.4E-05
Human toxicity, non-cancer effects	[CTUh]	1.1E-06	8.5E-07	1.0E-06	1.6E-05	1.6E-05	1.6E-05
Human toxicity (ReCiPe)	[kg 1,4-DB eq]	711.8	6.9	437.7	380.0	302.5	349.9
Particulate matter	[kg PM2.5 eq]	0.02	0.02	0.02	0.10	0.10	0.10
Ionising radiation HH	[kg U235 eq]	3.1	0.7	2.2	32.1	31.8	32.0
Photochemical ozone formation	[kg NMVOC eq]	0.4	0.3	0.4	3.0	3.0	3.0
Acidification	[molc H^+^ eq]	0.3	0.3	0.3	2.7	2.7	2.7
Acidification (EDIP)	[m^2^]	295.7	2.9	181.8	64.6	32.4	52.1
Terrestrial eutrophication	[molc N eq]	1.5	1.4	1.5	12.2	12.2	12.2
Freshwater eutrophication	[kg P eq]	0.0019	0.0011	0.0016	0.0539	0.0538	0.0539
Marine eutrophication	[kg N eq]	0.13	0.12	0.13	1.10	1.09	1.09
Freshwater ecotoxicity	[CTUe]	16.1	13.3	15.0	255.6	255.3	255.4
Mineral depletion	[kg Sb eq]	0.0001	0.0001	0.0001	0.004	0.004	0.004
Cumulative energy demand, non-renewable	[MJ]	313.5	65.0	216.9	3085.4	3058.1	3074.8

In the absence of fluorinated plastics, the PV could be treated through alternative processes (e.g. pyrolysis). This alternative scenario is analysed in Section 4.3.2.

The incineration of PV waste could be also be responsible for the emission of additional pollutants, including arsenic, cadmium, chromium and lead (Tammaro et al., 2016). However, no information was available on these emissions, so further research is recommended on this topic.

### 4.2 Impacts of PV recycling compared to other life cycle stages of the panel

LCA studies on PV technologies often exclude the EoL stage (Wambach, 2017). Previous studies also assumed that waste panels could be landfilled without any material or energy recovery (Battisti and Corrado, 2005). This section aims to assess how significant the impacts and benefits of the recycling process can be, compared to the other life cycle stages of the panel.

Although a detailed assessment of the whole life cycle of the PV panel is ‘out-of-scope’, this section presents a comparison of the impacts of the FRELP process (for c-Si PV panels with TPT backsheet) with the impacts due to manufacturing and potential benefits related to PV operation.

The impact of the manufacturing is based on inventory data from the literature (detail provided in the Supplementary materials, Section S.1).

With respect to the operation phase, the life cycle electricity output of the panels is estimated at around 208 MWh (detail provided in the Supplementary materials, Section S.1).

**Fig. 5:**
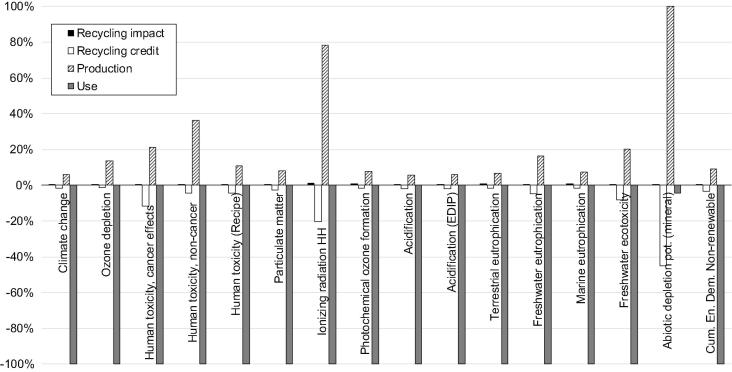
Comparison of the impacts and benefits of PV panels in different life cycle stages.

Fig. 5 shows the comparison of the impacts and benefits of the different life cycle phases of the PV panels, for 1 tonne of PV panels. The figure is built by assigning, for each impact category, the value of 100% to the life cycle stage responsible for the highest impact (or benefits); the impacts (or benefits) of the other phases are then represented proportionally. For example, in regard to climate change, the use phase is responsible for the highest benefit (in terms of CO_2_ eq emissions avoided), while the impacts of manufacturing for the same category are about 6%, and benefits of recycling are about 1% (impacts of recycling are negligible).

Fig. 5 shows that the use phase is the most relevant life cycle stage for the PV panel. The electricity produced during operation can be largely influenced by the initial assumptions (e.g. location and lifetime of the panels). However, a precise assessment of the impact of the use phase is not at the core of this article. Overall, benefits related to the generation of electricity are higher for any other benefit (or impact) during other phases, with the exception of ADP which is instead dominated by the manufacturing. Benefits from recycling are very significant for this impact category, granting a reduction of more than 40% of the impact of the manufacturing. Benefits from recycling are also significant (i.e. over 10%) for other impact categories, such as human toxicity (carcinogenic effects) and ionising radiation. The benefits of recycling for other impact categories range between 1% and 8% of those for the use phase, whereas impacts from recycling are always very low or negligible.

Results of this analysis could be used by LCA practitioners to improve the detail and comprehensiveness of their estimations concerning the EoL of PV panels.

### 4.3 Analysis of potential improvement scenarios for PV recycling

The previous sections investigated an innovative recycling process, as originally developed by industrial operators. Based on this analysis, we tried to identify potential areas for improvement. These focus, in particular, on optimisation of the overall logistics of the process (including transport between different facilities along the route) and potential alternative thermal treatments for the waste. Two new scenarios are introduced and discussed in the following sections.

#### 4.3.1 Decentralised treatment of PV waste

Analysis of the FRELP process proved that transport is one of the main aspects responsible for the impacts of PV recycling (Latunussa et al., 2016). Lunardi et al. (2018) concluded that transport of PV waste to the recycling facilities should be below 100 km for recycling being environmental convenient compared to other alternatives (e.g. direct incineration or landfilling).

In the analysis in the previous section, transport contributes to the impacts between 10% (for freshwater eutrophication) to 80% (for ADP). The high contribution from transport is related to the heterogeneous distribution of PV plants in the geographical areas, combined with the need to reach the specialised plant for recycling. Transport is also further affecting the incineration phase (step 6 in Fig. 1), since the scrapped PV sandwiches are first sent to the incinerator, then bottom ashes are sent back for further recovery of metals.

A potential improvement to the FRELP process could be achieved by decentralising the initial phases, with the creation of local plants for the pre-treatment of PV waste. Glass represents about 70% of the mass of panels, the metal framework and cables an additional 19%. A substantial reduction in the PV waste mass could be obtained by removing these elements in decentralised plants located close to PV waste collection points. Plants currently used for the collection and recycling of WEEE could be equipped with the technology for initial treatments in the FRELP process (steps 1 to 8 in Fig. 1). Subsequently, the panel’s scraps could be sent for thermal treatment (step 9 in Fig. 1). Finally, bottom ashes from incineration would be sent to the plant for the further FRELP treatments (steps 10 to 16 in Fig. 1). This solution has been indicated as technically feasible by the developers of the FRELP process. Economic viability of this strategy would depend on the amount of waste locally collected.

**Fig. 6:**
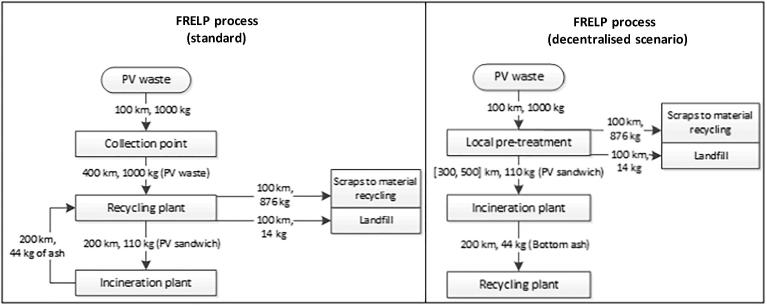
Comparison of transport in the FRELP process standard (as in Fig. 1) and in the improved scenario for decentralised treatments.

Overall, this scenario for decentralised treatment of PV waste entails a substantial reduction in transport (Fig. 6). The local pre-treatment allows a major reduction in the waste mass, while only remaining parts are sent to the incineration plant. The distance between the pre-treatment and the incineration plants is assumed variable, from 300 km to 500 km.

Table 3 shows the differences between this new scenario and the original FRELP scenario as in Section 4. The decentralised treatment could allow a significant reduction (i.e. larger than 10%) in the impacts for almost all the categories. In the case where waste is transported 300 km to the incineration plant, the reductions are particularly significant for impacts such as ADP, ozone depletion and human toxicity. In the scenario where it is transported 500 km, the benefits of the decentralised scenario are slightly lower.

**Table 3:** Impacts of scenarios for decentralised process.

Impact category	Unit	(A) FRELP	(B.1) FRELP decentralised	(B.2) FRELP decentralised	Variations (compared to scenario A)	
			(300 km)	(500 km)	B.1	B.2
Climate change	[kg CO_2_ eq]	4.6E+02	3.9E+02	4.0E+02	−15%	−14%
Ozone depletion	[kg CFC-11 eq]	3.3E-05	2.2E-05	2.2E-05	−34%	−31%
Human toxicity, cancer effects	[CTUh]	1.4E-05	1.0E-05	1.0E-05	−28%	−26%
Human toxicity, non-cancer effects	[CTUh]	1.6E-05	1.1E-05	1.1E-05	−32%	−30%
Human toxicity (ReCiPe)	[kg 1,4-DB eq]	3.5E+02	2.5E+02	2.6E+02	−28%	−26%
Particulate matter	[kg PM2.5 eq]	1.0E-01	8.1E-02	8.2E-02	−19%	−18%
Ionising radiation HH	[kg U235 eq]	3.2E+01	2.5E+01	2.5E+01	−22%	−21%
Photochemical ozone formation	[kg NMVOC eq]	3.0E+00	2.7E+00	2.7E+00	−10%	−10%
Acidification	[molc H^+^ eq]	2.7E+00	2.4E+00	2.4E+00	−10%	−10%
Acidification (EDIP)	[m^2^]	5.2E+01	4.9E+01	4.9E+01	−7%	−6%
Terrestrial eutrophication	[molc N eq]	1.2E+01	1.1E+01	1.1E+01	−8%	−8%
Freshwater eutrophication	[kg P eq]	5.4E-02	4.7E-02	4.8E-02	−12%	−11%
Marine eutrophication	[kg N eq]	1.1E+00	1.0E+00	1.0E+00	−9%	−8%
Freshwater ecotoxicity	[CTUe]	2.6E+02	1.9E+02	2.0E+02	−24%	−23%
Abiotic depletion potential (mineral)	[kg Sb eq]	4.4E-03	2.3E-03	2.4E-03	−47%	−45%
Cumulative energy demand, non-renewable	[MJ]	3.1E+03	2.0E+03	2.1E+03	−35%	−32%

#### 4.3.2 Use of fluorine-free backsheet combined with pyrolysis treatment

Another key aspect of PV recycling relates to the content of fluorinated plastics in the backsheet. Their thermal treatment must occur in dedicated plants provided with proper abatement systems for acid emissions (especially HF). On the other hand, the incineration of the PV waste must be separated from the treatment of other waste, in order to allow collection of the bottom ashes.

This incineration process entails some technical problems. Firstly, it is necessary to provide a certain ‘critical mass’ and continuity of waste as input, to sustain the incineration in the plant. This could represent a constraint to full development of the FRELP process, since the volume of PV panel currently reaching EoL is still very limited. Indeed, as discussed by Wambach (2017), the pilot development of the FRELP process was interrupted in spring 2016 because of the small volume of waste panels currently collected. Secondly, mass incineration of large amounts of PV scraps with fluorinated plastics could become problematic for the plant, which would risk exceeding legal limits for HF emissions. Finally, collection of the bottom ashes can be technically difficult and characterised by potential losses, which need to be further investigated.

Alternatively, fluorine-free panels could be treated by different plants. A new ‘pyrolysis scenario’ is therefore introduced. This supposes that fluorine-free PV waste (with PPE backsheet), after the pre-treatments (steps 1 to 8 in Fig. 1), is treated in a fixed-bed pyrolysis plant. This represents a new step in the process, in place of incineration (step 9 in Fig. 1). Residuals from the pyrolysis, also including valuable metals, could then undergo the next treatments similar to FRELP steps 10 to 16 (Fig. 1). Some additional details of the pyrolysis scenario are presented in the Supplementary materials (Section S.2).

Such scenarios could have multiple potential benefits. Firstly, the pyrolysis could be run in smaller plants (compared to incinerators), making it easier to reach the critical mass of waste input. The use of a pyrolysis plant would allow the PV waste to be treated in small batches, with easier collection of the bottom residual for recovery of metals, and with minor losses (compared to those occurring in a large incinerator). The pyrolysis could also occur in a plant located close to the pre-treatment, also allowing a reduction in the impacts caused by transport. The construction of a specialised pyrolysis plant for PV waste treatment would be easier (and less expensive) than a large incinerator with a plant for the abatement of acid gases. Finally, such a recycling process would be free from HF emissions, although this benefit is related to the composition of the waste and not to the pyrolysis itself.

Table 4 shows the impacts and benefits for the standard FRELP process (for PV waste with TPT backsheet), compared to those for the pyrolysis scenario. Pyrolysis is assumed to occur in the same facility as pre-treatments of the PV waste (avoiding the transport between step 8 and 10 in Fig. 1). All other steps are assumed to be the same.

**Table 4:** Comparison of the impacts of FRELP process with pyrolysis scenario.

Impact category		FRELP		Pyrolysis scenario	
		Impact	Benefit	Impact	Benefit
Climate change	[kg CO_2_ eq]	461.0	−2,398.3	361.3	−2,365.6
Ozone depletion	[kg CFC-11 eq]	0.00003	−0.00016	0.00004	−0.00016
Human toxicity, cancer effects	[CTUh]	0.00001	−0.00062	0.00001	−0.00062
Human toxicity, non-cancer effects	[CTUh]	0.00002	−0.00021	0.00001	−0.00021
Human toxicity (ReCiPe)	[kg 1,4-DB eq]	349.9	−5135.2	290.7	−5203.6
Particulate matter	[kg PM2.5 eq]	0.1	−1.4	0.1	−1.4
Ionising radiation HH	[kg U235 eq]	32.0	−586.4	30.4	−582.2
Photochemical ozone formation	[kg NMVOC eq]	3.0	−6.1	3.8	−6.0
Acidification	[molc H^+^ eq]	2.7	−15.1	2.7	−15.0
Acidification (EDIP)	[m^2^]	52.1	−214.7	32.5	−213.8
Terrestrial eutrophication	[molc N eq]	12.2	−22.5	12.0	−22.3
Freshwater eutrophication	[kg P eq]	0.054	−1.6	0.1	−1.6
Marine eutrophication	[kg N eq]	1.095	−2.0	1.1	−2.0
Freshwater ecotoxicity	[CTUe]	255.4	−8,055.6	246.3	−8,048.1
Mineral depletion	[kg Sb eq]	0.004	−4.8	0.004	−4.8
CED Non-renewable	[MJ]	3,074.8	−28,286.3	2,899.4	−28,863.9

The pyrolysis scenarios proved to have generally lower impacts than the standard FRELP process (Table 4), including climate change (20% reduction), human toxicity (ReCiPe; 20% reduction) and acidification (EDIP; 40% reduction). These benefits are mainly due to the absence of fluorine in the waste. On the other hand, pyrolysis has higher impacts for certain categories (e.g. photochemical ozone creation), due to some of the pollutants emitted (mainly carbon dioxide and carbon monoxide).

The benefits of the two processes are very similar. Compared to the standard FRELP process, the net benefits (i.e. benefits net of impacts) of the pyrolysis scenario are around 3% higher for climate change, human toxicity (ReCiPe) and CED. Benefits are 25% lower for photochemical ozone creation impact. However, emissions from pyrolysis were roughly estimated and direct measurements would be required for more precise estimation of the impacts. Moreover, parameters for the pyrolysis process (e.g. temperature and residence time) should be optimised according to the input PV sandwich waste.

It is argued by the developer that the pyrolysis scenario could be industrialised more easily, thus overcoming some of the barriers identified for the FRELP process. Obviously, the pyrolysis scenario is only theoretical and could be used only if recyclers were certain that no halogenated components were embodied in the PV panel. Aryan et al. (2018) proved that the pyrolysis of fluorinated backsheets is unfeasible both from an economic as well as technical viewpoint. For this purpose, the study by Fraunhofer (2017) suggested the potential labelling (or marking) of PV panels by manufacturers, according to the fluorine content. This labelling could allow EoL operators to identify fluorine-free waste and to sort it for optimised recycling (e.g. through pyrolysis).

## 5 Limitations of the analysis and further research needs

The previous sections analysed the resource efficiency of PV recycling processes, including some improvement scenarios. FRELP has been listed among the most innovative processes available so far (Wambach, 2017). However, this recycling process is still in its pilot stage. This represents the major limitation of the present study, as data on the input and output flows have been based on estimations and testing conducted by the designers of the FRELP process. More precise assessments could be performed once the process was industrialised and direct measurements were available (e.g. regarding energy consumed, emissions of pollutants, and quantities of materials recycled). Experimental data on incineration of the PV sandwich would be particularly useful, including measurements on the type and quantity of pollutants emitted. The scientific literature is currently lacking studies on this subject.

Another limitation of the study relates to information on the composition of the PV panels. Sample testing of PV waste has been conducted by the industrial developers of FRELP (SASIL, 2015, Latunussa et al., 2016). However, these data refer to PV waste panels currently collected, which were probably produced some decades ago. Analysis of the resource efficiency of PV recycling should be updated to reflect the evolved composition of the panels. Peeters et al. (2017) already identified certain trends in the material composition of PV, in particular declining silver content. This aspect can be highly significant, since silver is the main economic driver for the development of high efficiency recycling processes (SASIL, 2015). Low quantities of silver in future panels could discourage industrial investments in research and development for PV waste recycling. The monitoring of silver and other valuable materials (e.g. copper, aluminium and silicon) in the panel will be relevant for both researchers and policymakers.

Additional research is also required concerning the improvement scenarios, as discussed in Section 4.3. The two alternative scenarios have been judged feasible by the industrial developer of the FRELP process. However, their realisation necessitates further investigations. In the case of the decentralised treatment, a major bottleneck could be the economic viability of creating several small plants distributed across the territory. More precise forecasts of the quantities of PV waste generated in future (and their geographical distribution) would help to optimise collection and recycling strategies. The assessments in the previous sections did not take into account the impacts of capital equipment (i.e. impacts due to setting up the plants). We could expect a possible break-even point where the impacts from establishing several decentralised plants would overcome the benefits due to the reduced transport of waste.

Section 4.1.3 also showed the importance of accounting for the quality of SRMs produced through the recycling processes, as already recognised in the literature (Amini et al., 2007, Velis and Brunner, 2013, Iacovidou et al., 2019). This accounting is particularly important to provide insights into retaining the functionality and value of these materials, and how to enable their circularity in the economy (Iacovidou et al., 2017a). Assumptions on SRMs produced from the recycling of PV waste affect the environmental assessment, due to the selection of primary materials potentially being substituted by recycled materials. In our analysis, this selection was addressed through close cooperation with the industrial partners who developed the FRELP process. However, *ex post* analyses of the resource efficiency of PV recycling should in future be carried out based on documented uses of SRMs (mainly glass and silicon) recycled from the panels. Further research should take into account multidimensional values that span from other scientific domains (e.g. social, economic and technological) (Iacovidou et al., 2017b, Millward-Hopkins et al., 2018). It should also take a more comprehensive and holistic approach, to overcome the sectorial approach limitation in the LCA (Amini et al., 2007, Iacovidou et al., 2017b). It is emphasised here that the low quantity of PV waste currently collected represents the main limiting factor for further development and industrialisation of the FRELP process.

Scenario 4.3.2 was judged technically feasible by the industrial developer of FRELP and in fact this scenario was initially considered the preferred option, until the problems relating to fluorinated pollutant emissions were detected. However, further experimental tests should be carried out on the energy and material flows for pyrolysis, and on the suitability of the pyrolysis residues for the recovery of silver and silicon.

Finally, the labelling of fluorine-free PV panels is a pre-requisite for the scenario 4.3.2. However, this measure could only be applied to panels put on the market in future. This means that the sorting of fluorine-free panels could be practised only with a long time horizon (i.e. when labelled panels reach their EoL). Further research is also needed to demonstrate that the use of fluorine-free plastics can maintain or improve performance of the panel during operation.

## 6 Conclusions

The article assessed the resource efficiency, and related environmental benefits and burdens, of PV waste recycling processes. An innovative high-technology process (FRELP) was compared with current recycling processes used in European WEEE recycling plants (baseline process). An initial finding is that such an innovative process certainly meets the recycling targets set by European WEEE legislation, whereas the baseline processes have questionable capacity to reach such targets. The advantages of FRELP, compared to current recycling processes, are even more evident with regard to the recovery of silver and silicon (a CRM).

The differences between the environmental burdens and benefits of the two processes are worthy of comment. The FRELP process is characterised by higher impacts, but also higher benefits, for all the impact categories considered. Despite low recycling efficiency, the baseline process is still characterised by high environmental benefits, especially for climate change. This is mainly due to efficient recycling of aluminium in the baseline process. The benefits of the FRELP process are more evident when considering impact categories focusing on the use and recovery of raw materials (e.g. ADP).

Special attention was paid to air emissions during recycling and, in particular, during thermal treatments. The incineration step is essential to allow the further recycling of silver and silicon but, on the other hand, the presence of fluorinated plastics can be responsible for the release of toxic HF emissions. It was observed that various impact assessment methods commonly used in LCA are not capturing this type of emission. This highlights the need for LCA practitioners to carefully check that main process emissions are adequately characterised in the LCIA phase.

The article also confirms that the benefits of PV recycling are generally very low compared to the benefits of the use phase, with some exceptions (e.g. ADP).

A substantial reduction in the impact of the FRELP recycling process could be achieved with optimised logistics, based on local pre-treatment of PV waste followed by further treatment in a centralised plant. Even the potential treatment of the PV sandwich by pyrolysis could entail several benefits. However, pyrolysis would be applicable only for fluorine-free waste. Future labelling of PV panels (for new products put on the market) could help to sort fluorine-free panels for their optimal recycling.

The results also highlight some considerations for policymakers and PV manufacturers. Firstly, mass-based indicators for waste policy targets are relevant but do not encourage the efficient recovery of materials present in small traces (e.g. precious metals and CRMs). Dedicated targets (e.g. specific recycling targets for certain materials) could increase the efficiency of PV waste recycling.

It is also important to couple waste legislation with product design considerations. Here, future policy measures could facilitate better availability of data on the material composition of PV panels put on the market. These measures could be particularly relevant, for example, in terms of the content of silver (as the main economic driver for high-efficiency recycling processes) and halogenated plastics. It is also questionable whether PV panels with fluorinated plastics could be recycled at all through thermal treatments, as discussed in Section 4.3.2.

Finally, it is confirmed that the low quantity of PV waste collected so far is discouraging investments in industrial processes for PV recycling. However, this situation is not a justification for delaying research in this field, or the problem of managing PV waste is simply postponed to the near future. Claims about the sustainability of PV technologies cannot be fully supported until efficient and environmentally-friendly recycling processes for them have been developed and are deployable.

## Footnotes

1 TPT is considered as one of the most widely used materials for backsheet available on the market (Cancelliere and Liciotti, 2016). The type of backsheet can influence the recycling routes and overall efficiency of the recycling process (as discussed in Section 4.1.3).

4 The figure also takes into account losses in the production of SRMs: copper (3%), aluminum (3–5%), glass (10%).

5 Analysis based on direct observations and discussion with recycler. There is insufficient evidence on the effective amount of glass actually separated and whether its quality would be sufficient for functional recycling. The 10% value is a rough estimate. PV shredding residues could be incinerated, however with negligible energy recovery.

6 Lower heating value and fluorine content of the materials are (Fraunhofer, 2017): TPT – 26.6 MJ/kg, fluorine 5.5% in mass; KPK – 24.6 MJ/kg, fluorine 9% in mass; PPE – 30 MJ/kg, fluorine-free. The Ecoinvent (2007) database provided the information on the production of electricity and heat from plastics incineration.

## Footnotes

2 The inventory data underlying the background systems (including energy and material inputs) are derived from the Ecoinvent (2007) database and modelled through SimaPro software (Prè Consultants, 2018).

3 The impact assessment in Latunussa et al. (2016) mainly focused on the impacts recommended in the International reference Life Cycle Data system (ILCD) (EC, 2011), with the exceptions of ‘Mineral, fossil & renewable resource depletion’ (substituted by the impacts ‘Abiotic depletion, fossil’ and ‘Cumulative Energy Demand’) and the impacts on ‘land use’ and ‘water resource depletion’ (which were excluded).
