# Supplementary material for: Resource efficient recovery of critical and precious metals from waste silicon PV panel recycling
Source: Waste Manag. 2019 May 15;91:156–67. doi: 10.1016/j.wasman.2019.04.059 (PMC6591708; doi:10.1016/j.wasman.2019.04.059)
Supplement: Supplementary Data 1 [file mmc1.pdf]

Supplementary materials

Resource efficient recovery of critical and precious metals from waste silicon PV panel recycling

Fulvio Ardente, Cynthia E.L. Latunussa, Gian Andrea Blengini

Section S1. Details on impacts of PV manufacturing and energy production during operation

The impact of manufacturing is based on inventory data from the literature (information from the study by Jungbluth et al. (2009), as integrated into the Ecoinvent (2007) database). The dataset refers to production plants in Western Europe. The manufacturing phase included: production of the cell matrix; cutting of foils and washing of the glass; production of laminate and isolation; production of the aluminium frame; and treatment of production waste. Data were not available on direct air and water emissions from this process.

Energy production during operation of the PV panels has been estimated by the Photovoltaic Geographical Information System web application, developed by the European Commission Joint Research Centre (PVGIS, 2018). This application enables users to estimate the average monthly and annual energy production of a PV system, taking into account the characteristics of the PV modules and environmental parameters (including solar radiation, air temperature and wind speed). The user can also select details on how the modules are mounted. The analysis considered the PVGIS web application version 4, available until October 2018; a new version was expected to be released at the end of 2018.

Sections 4 and 5 considered the functional unit of 1 tonne of c-Si PV panels. Assuming average panels with 22 kg mass and 1.6 m^2 surface area (BioIS, 2011), 1 tonne of PV panels as considered in the functional unit will have an overall surface area of approximately 73 m^2. The PV panels are assumed to be installed on buildings located in northern Italy, and to have a 20-year lifetime, with 14 % system loss. Details of the input data are presented in Figure S.1. The result from the PVGIS web tool is that the panels would produce around 208 MWh of electricity over their lifetime. For the environmental assessment, it was estimated that this energy would replace electricity from the grid according to the Italian energy mix, referring to the dataset provided by Ecoinvent (2007).

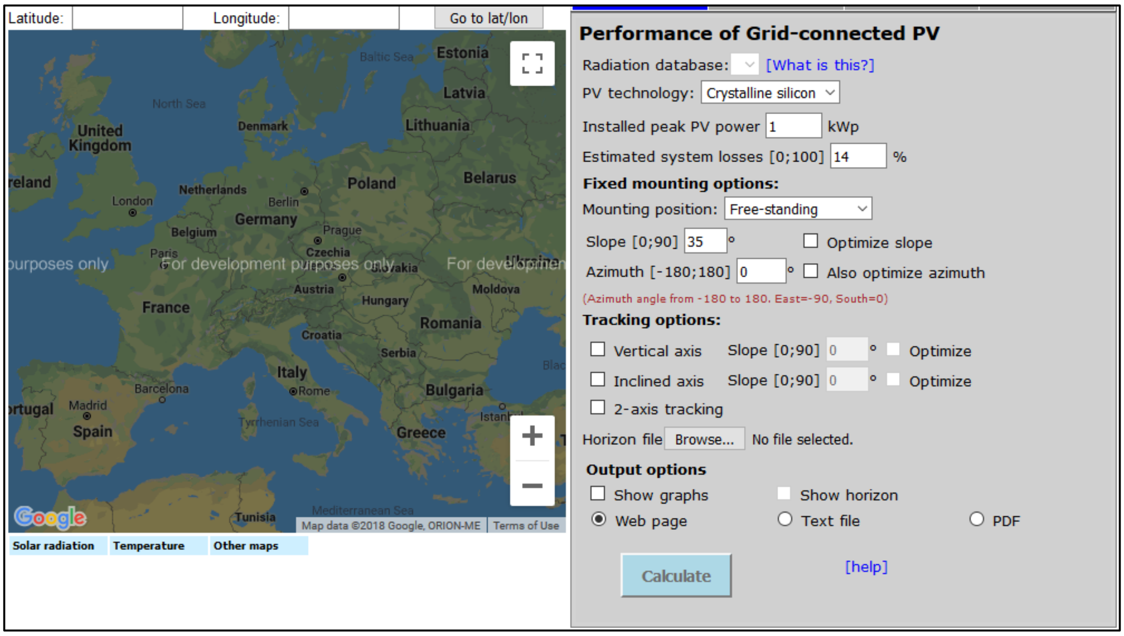

Figure S.1. Overview of PVGIS web application version 4, used to estimate the energy output of PV panels

Section S2. Additional detail on the pyrolysis scenario

The impacts of the pyrolysis scenario (as discussed in section 6.2) have been estimated based on Sharuddin et al. (2016). These authors investigated the result of pyrolysis processes for several types of plastic, with different technologies and temperature conditions. In our analysis we considered the results for pyrolysis of PET, which represents 71 % of the weight of PPE backsheet considered in the article. In particular, it was considered that pyrolysis of PET at 500 °C in a fixed bed reactor would yield two different phases: liquid (23 % by weight) and gas (77 %). The production of solid by-products was considered negligible.

In assessment of the pyrolysis scenario, the liquid phase was assumed to substitute an equal quantity of diesel oil.

Gases emitted through the pyrolysis process may include hydrogen, methane, ethane, ethene, propane, propene, carbon dioxide and carbon monoxide. Results by Williams and Williams (1999) have been used to estimate the composition of the gases produced through pyrolysis of PET. In the pyrolysis scenario (section 6.2), gas emissions were assumed to be composed mainly of carbon dioxide (59 %) and carbon monoxide (34 %); the remaining emissions (7 % by weight) were assumed to be combusted to substitute energy production from natural gas.

References

Bio Intelligence Service (BioIS), Study on photovoltaic panels supplementing the impact assessment for a recast of the WEEE directive – Final report, 2011.

Ecoinvent Centre, 2007. Ecoinvent Version 2, https://www.ecoinvent.org/database/older-versions/ecoinvent-version-2/ecoinvent-version-2.html, (accessed 20 September 2018).Fraunhofer UMSICHT. 2017. End-of-life pathways for photovoltaic backsheets. Report of Fraunhofer Institute. http://www.coveme.com/fraunhofer-report (accessed 2 August 2018)

Jungbluth, N., Stucki, M., Flury, K., Frischknecht, R., Büsser, S., 2012. Life Cycle Inventories of Photovoltaics. Report for the Swiss Federal Office of Energy SFOE. http://esu-services.ch/fileadmin/download/publicLCI/jungbluth-2012-LCI-Photovoltaics.pdf (accessed 09 October 2018)

Photovoltaic Geographical Information System (PVGIS) web application – version 4. Website: http://re.jrc.ec.europa.eu/pvgis/apps4/pvest.php?lang=en&map=europe (accessed 20 May 2018).

Sharuddin, D.S.A., Abnisa, F., Ashri, W.M., Daud, W., Aroua, M.K., 2016. A review on pyrolysis of plastic wastes. Energ. Convers. Manage., 115, 308-326, ISSN 0196-8904. https://doi.org/10.1016/j.enconman.2016.02.037.

Williams, P.T., Williams, E.A., 1999. Interaction of Plastics in Mixed-Plastics Pyrolysis. Energ Fuel 1999 13 (1), 188-196. DOI: 10.1021/ef980163x
